# AKT Pathway Genes Define 5 Prognostic Subgroups in Glioblastoma

**DOI:** 10.1371/journal.pone.0100827

**Published:** 2014-07-01

**Authors:** Anna Joy, Archana Ramesh, Ivan Smirnov, Mark Reiser, Anjan Misra, William R. Shapiro, Gordon B. Mills, Seungchan Kim, Burt G. Feuerstein

**Affiliations:** 1 Department of Neurology, Barrow Neurological Institute, St. Joseph's Hospital and Medical Center, Phoenix, Arizona, United States of America; 2 University of Washington, Tacoma, Washington, United States of America; 3 Department of Neurological Surgery, University of California San Francisco, San Francisco, California, United States of America; 4 Department of Mathematics and Statistical Science, Arizona State University, Tempe, Arizona, United States of America; 5 Department of Systems Biology, MD Anderson Cancer Center, Houston, Texas, United States of America; 6 Integrated Cancer Genomics Division, Translational Genomics Research Institute, Phoenix, Arizona, United States of America; 7 University of Arizona College of Medicine, Phoenix, Arizona, United States of America; NIH, United States of America

## Abstract

Activity of GFR/PI3K/AKT pathway inhibitors in glioblastoma clinical trials has not been robust. We hypothesized variations in the pathway between tumors contribute to poor response. We clustered GBM based on AKT pathway genes and discovered new subtypes then characterized their clinical and molecular features. There are at least 5 GBM AKT subtypes having distinct DNA copy number alterations, enrichment in oncogenes and tumor suppressor genes and patterns of expression for PI3K/AKT/mTOR signaling components. Gene Ontology terms indicate a different cell of origin or dominant phenotype for each subgroup. Evidence suggests one subtype is very sensitive to BCNU or CCNU (median survival 5.8 vs. 1.5 years; BCNU/CCNU vs other treatments; respectively). AKT subtyping advances previous approaches by revealing additional subgroups with unique clinical and molecular features. Evidence indicates it is a predictive marker for response to BCNU or CCNU and PI3K/AKT/mTOR pathway inhibitors. We anticipate Akt subtyping may help stratify patients for clinical trials and augment discovery of class-specific therapeutic targets.

## Introduction

WHO grade IV astrocytoma or glioblastoma (GBM) are the most common primary brain tumors and, unfortunately, the most aggressive. Median survival of patients harboring these tumors is approximately 14 months. Despite a committed effort to investigate new chemotherapies, molecularly targeted therapies, immunotherapies, surgical and radiological approaches, there has been little improvement over the last 30 years. Inadequate classification of GBM may have contributed to the difficulty of developing new therapies by decreasing power of clinical trials and underestimating benefit of class-specific drugs. It may also have confounded discovery of class-specific pathways and drug targets.

We know GBM diagnosed by histopathology is a collection of molecular and clinical subtypes. For example, there are two classes of GBM based on clinical presentation [1], [2]. Primary GBM arise de novo in older patients and are associated with poorer prognosis. Secondary GBM are rare (∼5-10% of total GBM), progress from lower grade tumors, occur more frequently in younger patients with better prognosis and have a different molecular profile. Studies using gene expression, DNA copy number, miRNA, and DNA methylation show these molecular characteristics can divide GBM into subclasses, some with different clinical characteristics [3], [4], [5], [6], [7], [8], [9]. Three subtypes emerged in early studies of WHO grade IV GBM (studies that combine histological subtypes or grades of glioma and use molecular classification to distinguish them are excluded from this discussion). These were called proneural (PN), Proliferative (PROLIF) and mesenchymal (MES) and each had characteristic clinical and molecular features [4]. Later approaches find 3–5 GBM subtypes including the PN, MES and Classical (CLAS) subgroups [8], [9], [10], [11]. DNA methylation identifies a subset of PN tumors with glioma CpG island methylator phenotype (GCIMP) that are younger, longer surviving and tightly associated with IDH1 mutations [8].

However, molecular classification of GBM is still in its infancy. There is no consensus on the number of subtypes and which classifiers best identify them. In addition, there is considerable reassignment of tumors to different classes depending on classifier used. We also have little information on which oncogenic pathways are active in subtypes and how subtypes respond to standard and experimental therapeutics. These questions need to be addressed before molecular classification can be reliably incorporated into clinical trials and patient treatment.

Alterations in the growth factor receptor/phosphatidylinositol 3-kinase/AKT (GFR/PI3K/AKT) pathway occur in most human cancers including at least 85% of GBM [10]. Pharmacological inhibition of the GFR/PI3K/AKT pathway is a promising strategy for anti-cancer therapy [12], [13]. However, while sporadic responses have been reported, clinical trials of pathway inhibitors in GBM have been largely disappointing [14]. Analyzing differences in pathway signaling among GBM subclasses may clarify and improve development and testing of these agents.

The GFR/PI3K/AKT pathway is complex and nonlinear having many inputs from other pathways [15], [16], [17], multiple sites of feedback regulation [18], [19], and a large number of downstream effectors [20]. Signaling within a pathway may depend upon cell state, history and environment. AKT is a key node in the pathway. We hypothesized that AKT pathway variation between tumors contributes to poor activity of inhibitors in clinical trials. We developed a list of genes associated with the pathway and asked whether their expression is sufficient to group GBM cases. Our data show AKT based subtyping gives at least five GBM subgroups with distinct molecular features and clinical courses. The evidence indicates AKT subtyping predicts response to a chemotherapy agent and GFR/PI3K/AKT pathway inhibitors.

## Materials and Methods

### Patient Information

The discovery dataset (GBM195) consisted of 181 GBM (WHO grade IV astrocytoma; (159 primary and 22 recurrent) from 3 datasets [3], [4], [21] and 14 non-neoplastic samples from 2 sources: (1) six samples from patients undergoing temporal lobe epilepsy surgery [3] and (2) eight samples from autopsy specimens of cerebral cortex from donors with no history of neurological disorders obtained from the National Neurological Research Brain Bank (Los Angeles, CA) [4]. Two datasets are in GEO (GSE 4271, GSE4412) and the third has been submitted. Table S1 lists GEO ID's and clinical information for GBM195 tumors. Tissue collection and processing, pathological review, and microarray analysis for the discovery dataset (GBM195) has been described elsewhere [3], . The validation dataset consisted of 583 samples; 573 GBM (16 recurrent and 3 secondary) and 10 non-neoplastic samples from The Cancer Genome Atlas (TCGA). Samples were collected and processed as described [5]. IRB or Committee on Human Research approval was obtained for samples used in the discovery and validation datasets as described [3], [4], [10], [21].

### Processing and analysis of microarray data

The PI3K/AKT pathway integrates information on cellular environment, energy status, stress and developmental stage to regulate apoptosis, autophagy, translation, metabolism, stem cell function and cell cycle [20], [22]. This involves multiple sites of crosstalk with other pathways. To capture the full function we generated a gene list that includes upstream and downstream gene products that directly or indirectly regulate or are regulated by AKT. This includes: (1) proteins or members of protein complexes that bind to, modify or regulate activity or subcellular localization of AKT (2) proteins or members of protein complexes phosphorylated or regulated by AKT, (3) proteins known to regulate or be regulated directly or indirectly by AKT (e.g. AKT through MDM2 regulates levels of TP53 protein). These genes were taken from: (1) a database of AKT interacting proteins (BOND [23]), (2) a database of AKT substrates (http://kinasource.co.uk/Database/substrateList.php) (3) evidence from Pubmed of phosphorylation by AKT (search term AKT, January 2008), (4) evidence from Pubmed that a gene regulates or is regulated by AKT either directly or indirectly (search term AKT, January 2008). Eliminating the genes with low variability across tumors within the discovery dataset left the 69 most variable genes used to classify AKT subgroups in the discovery dataset (Table S2). Five probes were not present in the validation dataset resulting in 64 of 69 AKT pathway genes applied during validation (Table S2).

We isolated patient subgroups in the discovery dataset using RMA normalized and median centered data [24]. We applied consensus k-means clustering with the Pearson's correlation coefficient as the similarity (1-distance) and complete linkage with 10,000 iterations using a sub-sampling ratio of 0.8. We then plotted the consensus distribution function (CDF) to find the optimal number of AKT subgroups [25]. Silhouette width values were computed for each sample [26] and only samples with a positive silhouette width were used in further analyses.

We isolated AKT subgroups in the TCGA validation dataset using raw data preprocessed as described for the discovery dataset. TCGA samples were mapped onto AKT subgroups in the discovery dataset by adapting the k means clustering algorithm. First, we found boundaries for each AKT subgroup in the discovery set by calculating the pairwise correlation coefficients between all samples within a subgroup. The minimum pairwise correlation coefficient was used as the lower boundary for each subgroup. TCGA samples were classified by computing the correlation coefficient between each TCGA and GBM195 sample. TCGA samples were assigned to an AKT subgroup if the average pairwise correlation coefficient with members of the group was greater than the lower boundary of that group. Ties were resolved by selecting the closest cluster.

### Analysis of GO terms

Conventional Gene Ontology (GO) enrichment analysis was dominated by generic GBM biological processes; therefore we used a single-sample approach analogous to the method used by Verhaak and Barbie [10]. To identify GO biological processes enriched within each individual sample we applied the hypergeometric test with Benjamini and Hochberg's correction on all expressed genes (using a two-fold change threshold from the median to determine up- and down-regulated genes). Neurodevelopmental terms enriched in > 20% of tumors were considered for analysis.

### Analysis of aCGH data

The GISTIC algorithm [27] was applied to the 456 TCGA samples with copy number information and results visualized using the Integrated Genomic Viewer (IGV) [28] to find copy number alterations (CNA) in the validation set. Broad copy number alterations in the discovery dataset were found as described previously [29] using a customized version of the Sanger CNV database [http://www.sanger.ac.uk/research/areas/humangenetics/cnv/]. For experiments that compare broad CNA in the discovery and validation dataset we identified broad copy number alterations in the validation dataset as follows. Briefly, we found the average q value (generated from the GISTIC algorithm) for 15 genes spaced evenly across the region of interest. If > 50% of genes had a q value less than expected by chance after correcting for multiple testing (q < 0.25), that region was called as a copy number alteration.

### Reverse Phase Protein Arrays

Level 3 (median centered, normalized, Z transformed) reverse phase protein array (RPPA) data was downloaded from the cBio Cancer Genomics Portal (http://www.cbioportal.org/public-portal/). One hundred and eighty six of the 215 tumors with RPPA data could be assigned to an AKT class and were used for analysis. Correlation coefficients between two antibodies against the same protein were high indicating adequate antibody specificity and pre-processing of data (Pearson correlation coefficient  =  0.83–0.98 for antibody pairs (GSK3A/B pS9/21, MAPK1, FOXO3, GATA3, S338 p-RAF1).

### Statistics

Differences between one subgroup and the rest were assessed using the F test for clinical variables and the likelihood ratio test for categorical variables. The Bonferroni method [30] was applied to correct for multiple hypotheses. We applied the Tukey HSD test to find pairwise differences between groups and correct for multiple comparisons [31]. Survival differences between subgroups were assessed using the Chi-squared test. Age was added to build a multivariate Cox model. For survival comparisons of BCNU/CCNU treatment between subgroups there were not enough observations to correct for age. After deleting all observations younger than 45, survival was no longer related to age. Significance was then determined using log rank. The Pearson goodness-of-fit test was used to assess the null hypothesis that proportions of G-CIMP tumors by subgroup and recurrent tumors by subgroup are equal to the proportions for all tumors by subgroup. P-values for these tests were calculated by Monte Carlo simulation since the counts of tumors by subgroup were too small to apply the large sample chi-square approximation. If the null hypothesis was rejected, then standardized residuals were used to determine which subgroups showed significant differences.

## Results

### AKT pathway gene expression divides GBM into at least six subgroups

**Figure 1:**
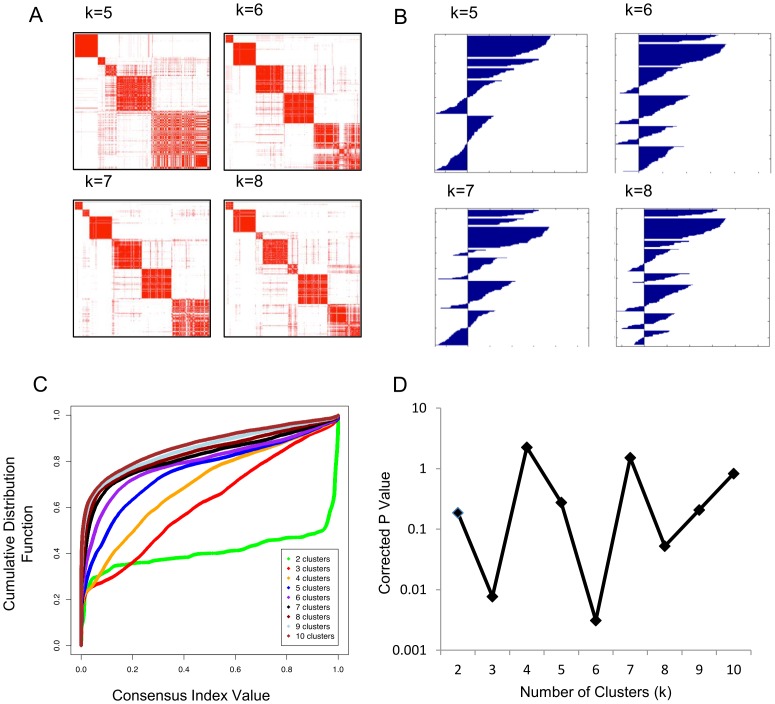
AKT pathway gene expression classifies GBM. (A) *Consensus heat maps for k = 5 to 8* generated with AKT pathway genes in the discovery dataset (GBM195). Red indicates total consensus (consensus index of 1) while white indicates no consensus (consensus index of 0). (B) *Silhouette scores for k  =  5 to 8* were calculated as described [26]. Samples with negative silhouette scores were removed in all further analysis. (C) *Consensus CDF for k  =  2 to 10*. (D) *Effect of k on survival differences between subgroups*. Kaplan Meier curves of patient subgroups were generated for k  =  2 to 10. For each k, Bonferroni corrected log rank p values were generated by pairwise comparison of subtypes. The smallest pairwise p value for each k is plotted.

We investigated AKT pathway variations in GBM by developing a list of AKT pathway genes (Table S2) then applying consensus clustering for the number of clusters k  =  2 to 10 (Figure S1; figure 1A shows results for k  =  5 to 8). We evaluated cluster stability using the consensus cumulative distribution function (CDF) plot of the consensus index (figure 1C) [25]. Cluster stability increased for k  =  2 to 6 but not appreciably for k > 6 (figure 1C); suggesting six is the optimum number of GBM AKT subgroups. Silhouette width values were computed for each sample [26] (figure 1B) and samples with a positive silhouette width were selected for further analyses.

We aim to have a classification system where clinical differences are maximized. Here, we investigated how survival of patient subgroups varies with k. Figure 1D plots the corrected p value between the longest and shortest surviving subgroups for each k. p values were low for k  =  3 and 6; k  =  6 was the lowest (figure 1D). This supports the CDF results selecting 6 clusters. The 6 consensus k-means subgroups were named cluster 1 (C1), proneural (PN), mesenchymal (MES), classical (CLAS), secondary-like (SL) and proliferative (PROLIF) based on their molecular and clinical features and prior naming [4], [32].

### Validation of AKT subgroups in an independent dataset

**Figure 2:**
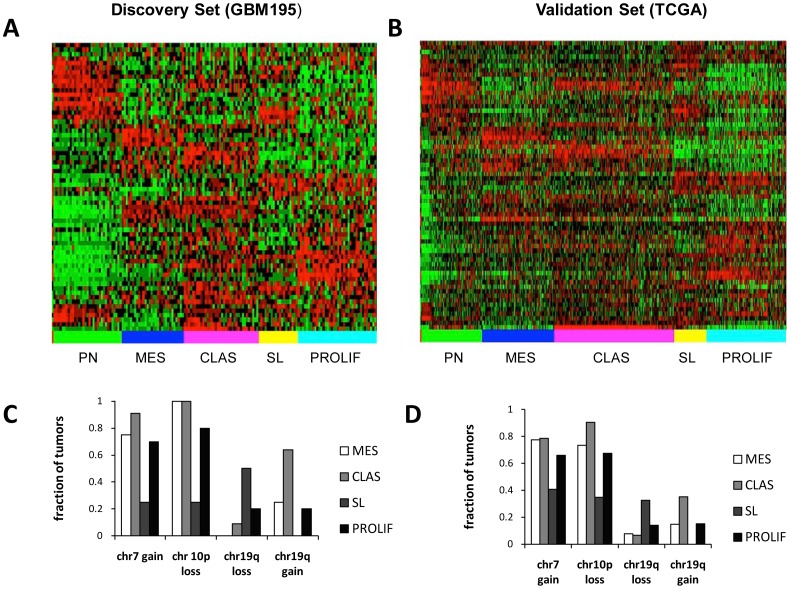
Validation of AKT subgroups in an independent dataset. *AKT pathway genes in discovery (A) and validation (B) datasets have similar patterns of expression in subgroups*. Tumors in the discovery and validation set were first grouped by AKT subgroup membership then ordered by correlation coefficient. AKT gene order in the discovery set was determined by one-way hierarchical clustering and retained in the validation set. *Discovery (C) and validation (D) datasets have similar DNA CNA*. The percentage of patients in the discovery (A) and validation (B) datasets with copy number gains or losses in chr7, 10 and 19q is shown.

We next validated AKT subgroups in an independent dataset of non-overlapping samples. TCGA samples were mapped onto discovery AKT subgroups by assigning a sample to the closest Akt subtype, as described in the methods section. Only two samples were assigned to AKT subgroup C1, therefore this subgroup was dropped from all further analysis. Figure 2 compares AKT pathway gene expression in the discovery (figure 2A) and validation (figure 2B) sets. It shows the pattern of expression of AKT pathway genes within subgroups is similar in both datasets. Interestingly, the PN subgroup in both datasets contained all non-neoplastic samples (not shown). We examined expression of AKT pathway genes in subgroups (Figure S2). These data show AKT classes arise from complex patterns of gene expression in subgroups. It did not point to a role for a specific part of the AKT pathway within any subgroup.

We next investigated correspondence between copy number alterations (CNA) in AKT subgroups from discovery (figure 2C) and validation (figure 2D) datasets. The PN subgroup was omitted since it had no CNA information in the discovery dataset. CNA within subgroups were similar in the discovery and validation datasets: a high percentage of tumors with 7gain/10 loss occurred in every subgroup except SL, the SL subgroup had greater frequency of 19q loss and the CLAS subgroup had increased gain of chr19q relative to the rest. Therefore all subgroup-associated trends in CNA within the discovery dataset were recapitulated in the validation dataset.

### TCGA, Phillips and G-CIMP subgroups distribute non-randomly in AKT subgroups

**Figure 3:**
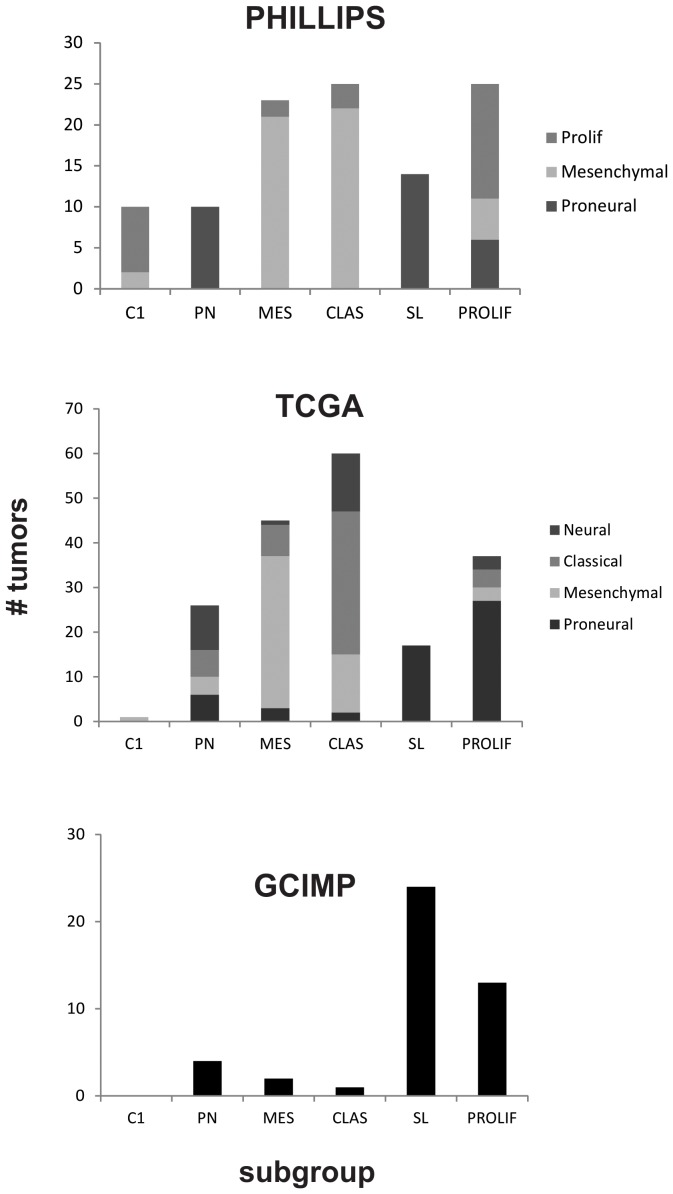
Previous classification systems distribute non-randomly in AKT subgroups. Distribution of Phillips (A), TCGA (B) and G-CIMP (C) subgroups in AKT subgroups.

Phillips, TCGA and G-CIMP subgroups distributed non-randomly in AKT subgroups (Figure 3A, B and C; Tables S3 and S4). There was a tendency for AKT subtyping to split each Phillips subgroup in two. The AKT PN and SL subtypes were significantly enriched in the Phillips PN subtype (Figure 3A, Table S3; p < 0.5 Bonferroni corrected). The AKT MES and CLAS subtypes were significantly enriched in Phillips MES subtype (figure 3A, Table S3, p < 0.5; Bonferroni corrected). The AKT PROLIF subtype was significantly enriched in the Phillips PROLIF subtype (Figure 3A, Table S3; p < 0.5; Bonferroni corrected). The enrichment of Phillips PROLIF tumors in AKT C1 subtype did not reach significance. AKT subgroups had less concordance with TCGA subgroups [10]. AKT SL and PROLIF subtypes were significantly enriched in TCGA PN subtype; while AKT MES and CL subgroups were enriched in the TCGA MES and CL subtype, respectively (Figure 3B, Table S4; p < 0.5; Bonferroni corrected). The AKT PN subtype was a mixture of all the TCGA subgroups. The AKT SL and PROLIF subgroups contained the majority of G-CIMP tumors (figure 3C). Taken together these data show AKT classification divides existing subgroups further.

### Patients in the SL subgroup are younger and have longer survival

**Figure 4:**
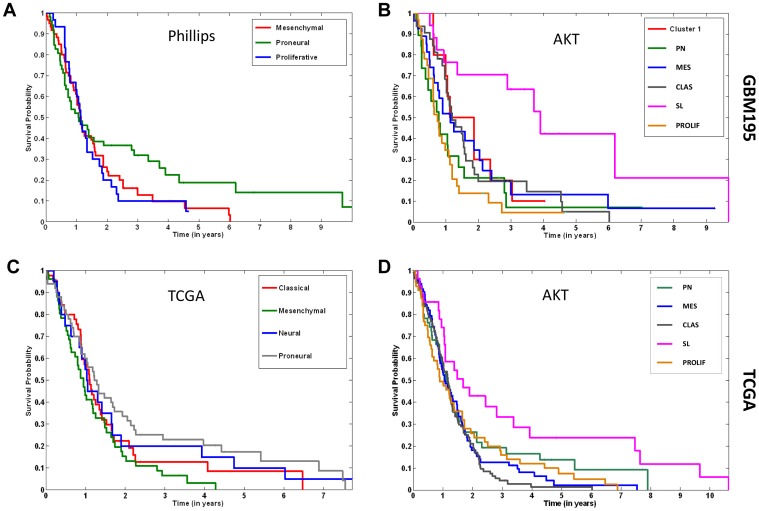
AKT subgroups are prognostic. Kaplan Meier survival curves plotted for Phillips (A) and AKT (B) subgroups in the discovery dataset and for TCGA (C) and AKT (D) subgroups in the validation dataset. Log rank p value  =  0.0005 (B; SL vs. rest); 0.0029 (B; PROLIF vs. rest) and 0.003 (D; SL vs rest). Survival differences did not reach significance in (A) and (C).

AKT subgroups have different clinical characteristics (figure 4B and D; Table S3 and S4). SL patients in the discovery dataset had longer median survival (3.9 vs. 1.05 yrs.; p  =  0.0005; figure 4b; SL vs. the rest) and were younger (median age  =  38 vs. 49; SL vs. total; p  =  0.05 using Tukey HSD test to correct for multiple comparisons; Table S3). After adjusting for age in Cox multivariate analysis, SL status remained a significant predictor of survival (p  =  0.027; SL vs. the rest). The PROLIF subgroup had statistically significant shorter survival than the rest (0.75 vs. 1.25 yrs.; p  =  0.0029; figure 4B) although age of these patients was not different than all patients (median age  =  49 vs. 49 years; PROLIF vs. total; Table S3). Although the magnitude was diminished, a similar trend was observed for SL patients in the validation dataset for survival (1.67 vs. 1.1 yrs.; p  =  .003 SL vs. rest; figure 4D) and age (median age  =  49 vs. 59 yrs.; p  =  .07; SL vs. total, Table S4) although the age difference was not statistically significant. In comparison, patient subgroups defined using Phillips (figure 4A) and TCGA (figure 4C) methods using the same database have no statistically significant differences in survival.

Consistent with the less aggressive character of SL tumors, there was a trend toward decreased endothelial proliferation (46% vs. 66%; p  = 0.017 vs. rest; uncorrected), and palisading necrosis (10% vs. 51%; p  =  0.07 vs. rest; uncorrected) in the validation dataset (Table S4). There were similar trends in the discovery set although they also did not reach significance (Table S3). Taken together these data show subgroups in the discovery and validation datasets have similar clinical features. It also shows AKT subtypes have distinct clinical characteristics.

### Evidence AKT subtyping is a predictive marker for sensitivity to BCNU/CCNU

**Figure 5:**
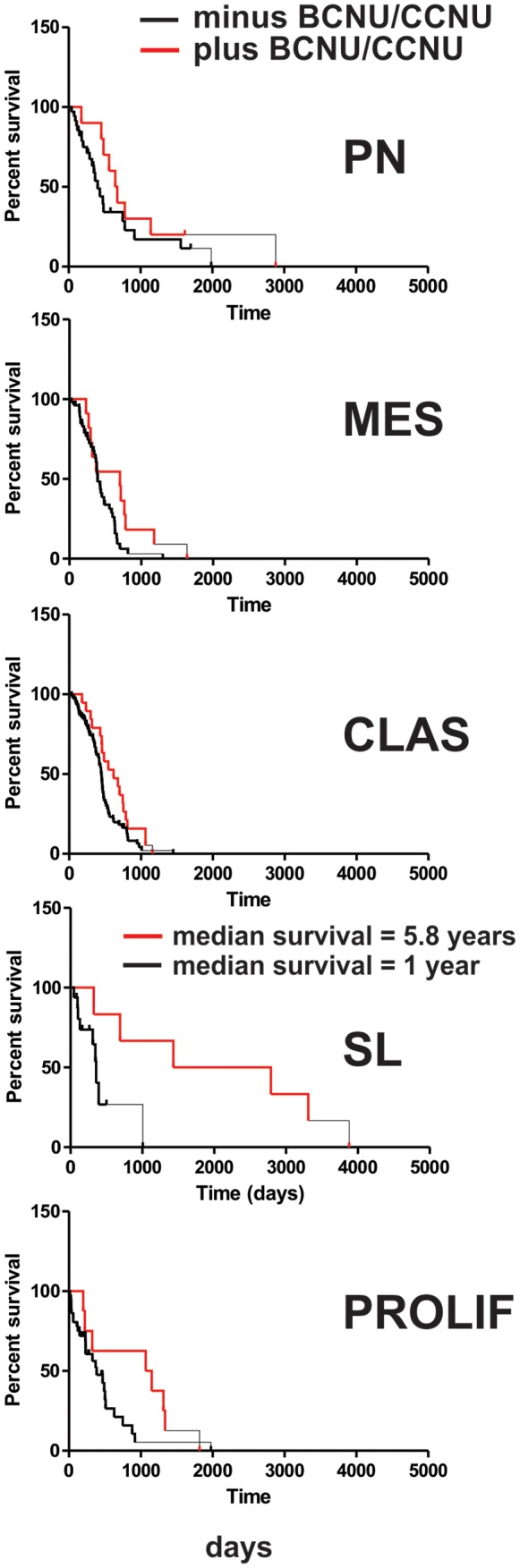
Evidence that SL patients are sensitive to BCNU and CCNU. Kaplan Meier survival curves for TCGA patients receiving (solid line) or not receiving (dashed line) alkylating agent (BCNU and/or CCNU) by subgroup. p  =  0.03 after correcting for age (SL subtype; log rank). n  =  6 and 16 for SL patients receiving or not receiving BCNU/CCNU, respectively.

Survival differences between subgroups suggest AKT subtypes are either prognostic or predictive (forecasts tumor aggressiveness or response to therapy, respectively). Since AKT influences response to chemotherapy [33], we hypothesize AKT subgroups are predictive markers. Indeed, TCGA SL patients treated with BCNU or CCNU had longer median survival than those receiving other treatments (figure 5; median survival  =  5.8 vs. 1.05 years; p  =  0.03 after correcting for age; log rank). Those receiving BCNU or CCNU were older and had less IDH1 mutations than those that didn't (median age  =  54 vs. 49 years; % with IDH1 mutations  =  17% vs. 32%; with vs. without BCNU/CCNU respectively); indicating age and IDH1 mutation status do not account their increased survival. This finding indicates patients in the SL subgroup are sensitive to BCNU and CCNU.

### Subgroups have distinct genomic alterations

**Figure 6:**
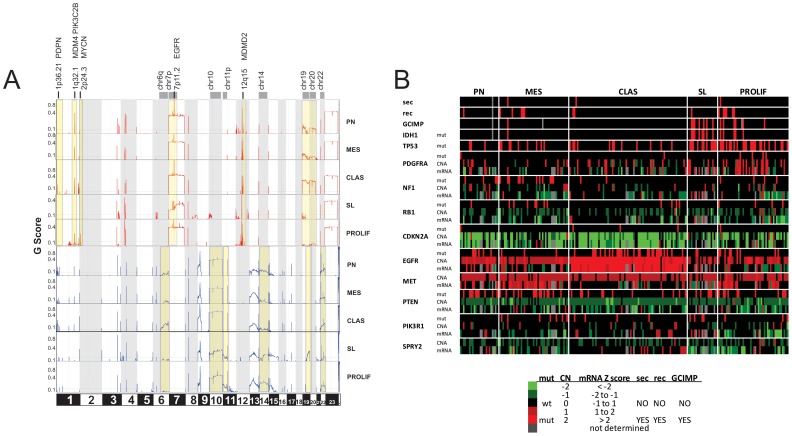
AKT subgroups have distinct genomic alterations. (A) *Copy number alterations in TCGA AKT subgroups*. The GISTIC method was applied to TCGA samples in each subgroup with copy number information. Data are presented as a G score which is an integrated score of the prevalence of the copy-number change times the average (log2-transformed) amplitude. The green line shows significance threshold (FDR q values to account for multiple-hypothesis testing). Regions with subgroup-specific CNA are highlighted in yellow. (B) *Distribution of clinical information and mutations, CNA and mRNA expression for glioma-associated genes in AKT subgroups*. The 218 TCGA GBM cases with gene expression, consensus putative copy number alteration and validated mutation data [55], [56] was used for this analysis (The cBio Cancer Genomics Portal; http://www.cbioportal.org/). Gene expression is represented as z scores calculated relative to diploid tumors for each gene and are the median value of 3 mRNA platforms (Affymetrix U133A and Exon arrays and Agilent custom array). There was a statistically significant enrichment of IDH1 mutations in the SL and EGFR and CDKN2A mutations plus CNA in the CLAS subtype (p < 0.02).

We used TCGA data to investigate how molecular alterations partition in subgroups. All subgroups had unique broad (figure 6A; Figure S3) and/or focal (Table S6 and S7) DNA CNA. The CLAS subtype was enriched in broad CNA previously associated with more aggressive tumors such as loss of chromosome regions 6q and gain of 19q and 20q [34](figure 6A). The SL subtype was enriched in broad CNA associated with better prognosis (loss of 19q; figure 6A) [34]. Each subgroup had unique focal CNA (Table S6 and S7). This data shows AKT subtyping groups tumors with similar molecular characteristics.

An integrated analysis of mutations, CNA and mRNA expression in glioma-associated genes shows some AKT subgroups had similar features as TCGA subgroups (figure 6B). The AKT CLAS subgroup was significantly enriched in alterations in EGFR and CDKN2A similar to TCGA CLAS subgroup [10]. The AKT MES subtype was characterized by mutations in NF1 and RB1 and increased mRNA for the mesenchymal marker, MET, similar to the TCGA MES subgroup [10], although these did not reach statistical significance. The SL subtype was enriched in IDH1 mutations (42% vs 3% SL vs. rest) and GCIMP (47% vs. 4%; SL vs. rest) although only the enrichment in IDH1 mutant tumors was significant. The PROLIF subtype was also slightly enriched in IDH1 mutations (11%) in this dataset containing 218 validated samples. However that dropped to 7% when considering all TCGA tumors with IDH1 mutation information (not shown). Both the SL and PROLIF subgroups were also enriched in alterations found more frequently in secondary tumors including TP53 mutations and increased mRNA and CN gains for PDGFRA. The PROLIF was distinguished from SL subtype by an increase in mutations and copy number alterations in EGFR and CDKN2A (figure 6B) and enrichment in recurrent tumors (18% vs 8%; PROLIF vs rest; Table S4). Genomic alterations in other RTK/RAS/PI3K/AKT pathway members were either not significantly enriched in any subgroup (PTEN, PIK3R1, MET, SPRY2; figure 6B) or the frequency was too low to evaluate (ERBB2, KRAS, NRAS, HRAS, PIK3CA, FOXO1, FOXO3, AKT1, AKT2, AKT3; not shown); although MET mRNA was enriched and SPRY2 mRNA was low in the MES and CLAS subtypes, respectively (figure 6B). Taken together these data suggest involvement of oncogenic and tumor suppressor pathways can differ between subgroups.

### Subgroups have distinct patterns of expression for PI3K/AKT/mTOR components

**Figure 7:**
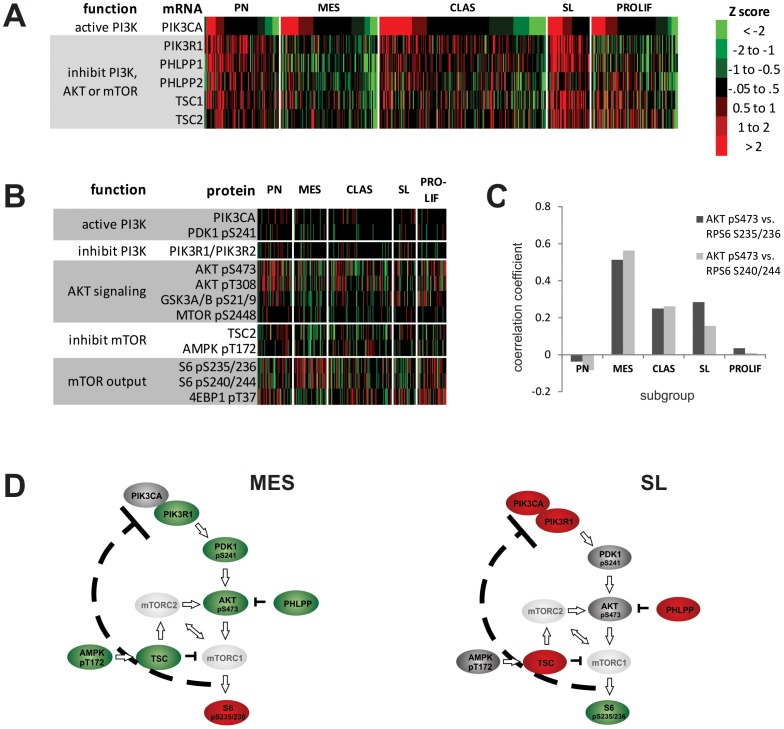
Subgroups have distinct patterns of expression for PI3K/AKT/mTOR pathway components. Tumors (x axis) were grouped by AKT class then Z transformed mRNA (A) or protein and phospho-protein expression (B) color coded to reflect magnitude (y axis). The Pearson correlation coefficient for AKT pS473 vs. RPS6 pS235/236 (light gray) and AKT pS473 vs. RPS6 pS240/244 (dark gray) for each subgroup is shown (C). Proposed AKT/mTOR/S6 pathway map for the MES and SL subtypes based on this data (D). This model shows loss of AKT and mTOR inhibitors (PHLPP, TSC and pAMPK) increases output of the AKT/mTOR/S6 axis (pRPS6) in the MES subgroup. Conversely, increased expression of these inhibitors decreases output in the SL subgroup. Red, grey and green represent high, intermediate and low expression/activity, respectively.

We find subgroups have distinct patterns of expression of mRNA (figure 7A), protein and phospho-proteins (figure 7B) for PI3K/AKT/mTOR pathway components. The most notable patterns were in the MES and SL subgroups. The MES subtype had decreased expression for inhibitors of mTOR, AKT and PI3K (TSC2 and p-AMPK protein; TSC1, TSC2, PHLPP1, PHLPP2 and PI3KR1 message). Consistent with increased activity of the AKT/mTOR/S6 axis, this subgroup also had elevated p-S6 (figure 7B) and a high positive correlation between p-AKT and p-S6 (figure 7C). The long surviving SL subgroup had the opposite pattern of expression; high expression of AKT and mTOR inhibitors (figure 7A and B), decreased expression of pS6 (figure 7B) and lower correlation between pAKT and pS6 (figure 7C). Our proposed pathway map for the MES and SL subgroups (7D) based on this data posits how expression of pathway inhibitors affects output of the AKT/mTOR/S6 axis. This data indicates subgroups will have different sensitivities to pathway inhibitors.

### GO terms suggest subgroups have a different dominant biological process and cell of origin

**Figure 8:**
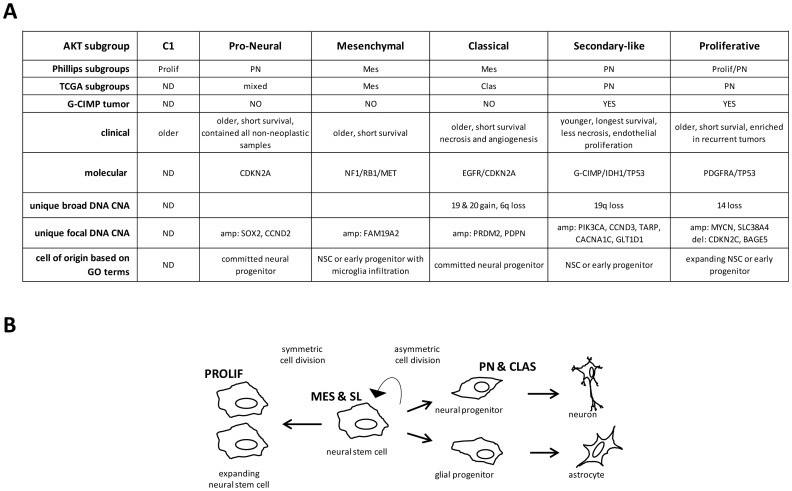
Summary of features in AKT subtypes. Clinical and molecular features of AKT subgroups are summarized in (A). Illustration of proposed neurodevelopmental cell of origin for AKT subgroups based on GO terms (B). ND  =  not determined.

We used Gene Ontology (GO) to investigate the biological role of genes expressed in tumors and how terms partition in subgroups. Each subgroup, except CLAS, had a high percentage of tumors with functionally related terms that suggested a different dominate biological process (Table S5). The CLAS subgroup had a mixture of terms. Each subgroup also had GO terms associated with neurodevelopment (Table S5; highlighted dark grey; summarized in figure 8B). The PN and CLAS subgroups had only terms associated with neurogenesis suggesting a committed neural precursor cell of origin. The MES, SL and PROLIF subgroups had terms associated with both neuro- and glio-genesis suggesting a stem cell or early uncommitted progenitor cell of origin. These data suggest the cell of origin and dominant biological process can differ in subgroups.

## Discussion

The major finding is that AKT pathway genes classify GBM into at least five patient subgroups with unique clinical and molecular characteristics. The results were validated in an independent dataset of non-overlapping samples, suggesting AKT classes reflect underlying structure in the data and do not arise from chance or technical artifacts such as batch effects and patient sampling. Taken together these data add to previous results suggesting histopathologically diagnosed GBM is a collection of molecular subgroups with fundamental differences in biology and clinical behavior. This approach advances classification of GBM by splitting out groups not previously identified by other approaches and expands our understanding of molecular aberrations underlying subgroups.

We interpret with caution the finding that SL patients treated with BCNU or CCNU have appreciably longer survival than SL patients receiving other treatments (median survival 5.8 vs. 1.05 years respectively). Inhomogeneity between the cohorts (including treatment protocols and institution providing tumor) could impact survival. However, age and IDH1 mutation status clearly do not contribute since patients in the longer surviving cohort were older and had less IDH1 mutations. If validated these results suggest AKT classification is a predictive marker that identifies a subset of GBM patients with sensitivity to BCNU/CCNU. Interestingly, there is a subset of anaplastic oligodendroglial tumors characterized by 1p19q loss of heterozygosity (LOH) and IDH1 mutations that significantly benefits from procarbazine, CCNU, and vincristine (PCV) chemotherapy [35]. This anaplastic oligodendroglial subtype shares similarities to the AKT SL subgroup (19q loss and IDH1 mutant tumors).

Mutations in IDH1 are a common and early event in low grade glioma, they are present in secondary GBM [36], [37], [38] and may cause the G-CIMP phenotype [36], [37], [38], [39]. One third of SL tumors have IDH1 mutations and CIMP. This subgroup also has other molecular similarities to secondary tumors (enriched for genomic alterations in TP53 and PDGFRA), longer survival and a tendency for less endothelial proliferation and pallisading necrosis (Tables S3 and S4). These data suggest tumors in the SL subtype are grade IV secondary tumors or borderline grade III/IV secondary tumors progressing to GBM. If this is true then genomic alterations associated with the SL subtype might be used as markers of progression for grade II/III secondary tumors. These results also indicate there is a population of GBM without IDH1 mutations that share clinical characteristics and a similar pattern of AKT pathway gene expression with the IDH1 mutant tumors. This suggests other paths beside IDH1 mutation give rise to the IDH1 mutant/CIMP phenotype.

We found distinct patterns of expression for PI3K/AKT/mTOR components in subgroups. Our results suggest gene products that inhibit AKT and mTOR are important regulators of PI3K/AKT/mTOR/S6 axis output. In our model the loss of AKT and mTOR inhibitors (PHLPP, TSC and pAMPK) increases output of the AKT/mTOR/S6 axis in the MES subgroup. Conversely, increased expression of these inhibitors decreases output in the SL subgroup. In an apparent paradox, p-AKT expression is low in the MES subgroup. We suggest AKT phosphorylation is held in check in the MES subgroup by (1) heightened activity of an mTOR/S6K/IRS1 negative feedback loop [40], [41], [42], [43] and (2) low TSC1 and 2 expression that decreases mTORC2 activation and AKT phosphorylation [42], [44]. Our model suggests the MES subtype will be sensitive to joint inhibition of mTOR and PI3K, but inhibition of mTOR alone will increase p-AKT. Interestingly, NF1 loss drives mTOR/S6 hyper-activation via AKT [45], [46], [47] and the MES subtype is enriched for NF1 loss. These data suggest subgroups have variations in AKT pathway signaling that will affect sensitivity to pathway inhibitors.

How do these results compare with other approaches that use mRNA to classify GBM? AKT classification is complementary to previous classification methods but divides GBM into more subgroups. It gives patient subgroups with statistically significant differences in survival while Phillips [4] or TCGA [10] methods do not when using the same database. The performance of the validation dataset is typically not as robust as the discovery dataset [48] and this may contribute to our inability to replicate survival differences seen by Phillips et.al. [4]. Interestingly, there was higher concordance between AKT classification and classification based on survival-associated mRNA used by Phillips et. al. [4] than most variable mRNA used by Verhaak et. al.[10]. We suggest classification schemes based on mRNA relevant to tumorigenicity, like survival-associated and AKT pathway genes, are more effective at partitioning tumors into clinically and molecularly relevant groups.

Survival differences found in the discovery dataset were diminished in the validation dataset. Inhomogeneity's between datasets that could confound comparisons including (1) age (median age  =  49 yrs. in discovery vs. 59 yrs. in validation dataset), (2) patient populations (three institutions contributed tumors to the discovery and eighteen to the validation dataset), (3) treatment (there were large variations in treatment regimens in the validation dataset). In addition, performance of the validation dataset is typically not as robust as the discovery dataset [48].

One AKT subgroup was not found in the validation dataset (C1). We know morphological heterogeneity can result in inconsistent intra- and inter-observer diagnosis of grade and histological type (astrocytoma, oligodendroglioma and mixed oligoastrocytoma) [48], [49], [50]. Therefore C1 may be a histological variant diagnosed as GBM and included in the discovery, but not the validation dataset.

GO term analysis suggests different cells of origin/dominant biological processes for each subgroup (summarized in figure 8A and B). The younger, longer surviving, SL patient subgroup with molecular similarities to secondary GBM had terms associated with both neuro- and glio-genesis suggesting a NSC cell of origin. Indeed, the longer survival of these patients is consistent with the quiescent nature of NSC. PROLIF tumors also contained neuro- and glio-genesis terms in addition to terms related to mitosis, spindle formation and cell cycle checkpoint. Literature suggests the balance between symmetric and asymmetric cell divisions regulates NSC [51] which is influenced by proteins with a role in spindle formation and mitotic progression [52]. Based on this and their aggressive nature we propose PROLIF tumors are derived from NSC with enforced symmetric cell divisions that rapidly expand the population (figure 8B). The ability of AKT classification to group tumors by cell of origin would suggest a major role for the PI3K/AKT pathway in neurodevelopment. This is consistent with reports showing a role for pathway members in NSC maintenance [53], [54].

We suggest AKT-based classification will augment drug development on many levels. This work indicates evaluating new drugs using all GBM patients combined with different natural courses and/or response to therapy can confound clinical trials. It suggests incorporating AKT classification will improve clinical trial design, decreasing their cost and maximizing the number of therapeutics that can be evaluated. In addition, AKT based classification may enhance drug discovery since new pathways and drug targets will be easier to find in molecularly homogeneous samples. We propose that robust molecular classification of GBM could ultimately improve patient care by guiding therapeutic planning, sparing patients ineffective treatments, both standard and experimental, and focusing on strategies more likely to work.

## Supporting Information

Figure S1 **Consensus k-means heat maps for k  =  2 to 10 generated with AKT pathway genes in the discovery dataset (GBM195).** Red indicates total consensus (consensus index of 1) while white indicates no consensus (consensus index of 0).
(TIF)

Figure S2 **Average expression of AKT pathway genes in subgroups.** Hierarchical clustering using AKT pathway genes was used to group GBM patients and genes in the discovery (GBM 195) dataset. Tumors in the validation dataset were grouped by AKT class keeping the same order of genes. The expression of AKT pathway genes in each class was averaged and is shown as a heatmap; red and green is high and low expression respectively.
(TIF)

Figure S3 **Log2 (tumor/normal) DNA copy number in subgroups.** Amplifications (red) and deletions (blue) in subgroups (y axis) were determined by segmentation analysis of normalized signal intensities from TCGA SNP arrays using GISTIC and viewed with IGV by chromosomal location (x axis).
(TIF)

Table S1 **Clinical information for tumors in GBM195.**
(XLSX)

Table S2 **AKT pathway gene classifiers used for the discovery and validation datasets.**
(XLSX)

Table S3 **Distribution of clinical and molecular information by subgroup in the discovery dataset (GBM195).** The table lists the number of tumors with the specified feature in each subgroup in the discovery dataset. Features with statistically significant enrichment in a subgroup after Bonferroni correction (p < 0.05) are highlighted.
(XLSX)

Table S4 **Distribution of clinical and molecular information by subgroup in the validation dataset (TCGA).** The table lists the number of tumors with the specified feature in each subgroup in the validation dataset. Features with statistically significant enrichment in a subgroup after Bonferroni correction (p < 0.05) are highlighted in dark grey. Features with statistically significant enrichment in a subgroup before Bonferroni correction are highlighted in light grey.
(XLSX)

Table S5 **GO term analysis of genes differentially expressed in subgroups.** The % of individual tumors within a subgroup that are enriched for a specific GO term is shown, ordered by decreasing representation. GO terms enriched in ≥ 40% of tumors are highlighted light grey. Neurodevelopmental terms are highlighted with dark grey.
(XLSX)

Table S6 **Focal DNA amplifications in subgroups.** Copy number alterations in subgroups were evaluated using GISTIC and the q score for statistically significant focal DNA copy number gains (q score < 0.25) listed. Focal copy number changes common to all subgroups (q < 0.25 in all subgroups) are not reported.
(XLSX)

Table S7 **Focal DNA deletions in subgroups.** Copy number alterations in subgroups were evaluated using GISTIC and the q score for statistically significant focal DNA copy number losses (q score < 0.25) are listed. Focal copy number changes common to all subgroups (q < 0.25 in all subgroups) are not reported.
(XLSX)
