# Supplementary figures and images for: AKT Pathway Genes Define 5 Prognostic Subgroups in Glioblastoma
Source: PLoS One. 2014 Jul 1;9(7):e100827. doi: 10.1371/journal.pone.0100827 (PMC4077731; doi:10.1371/journal.pone.0100827)

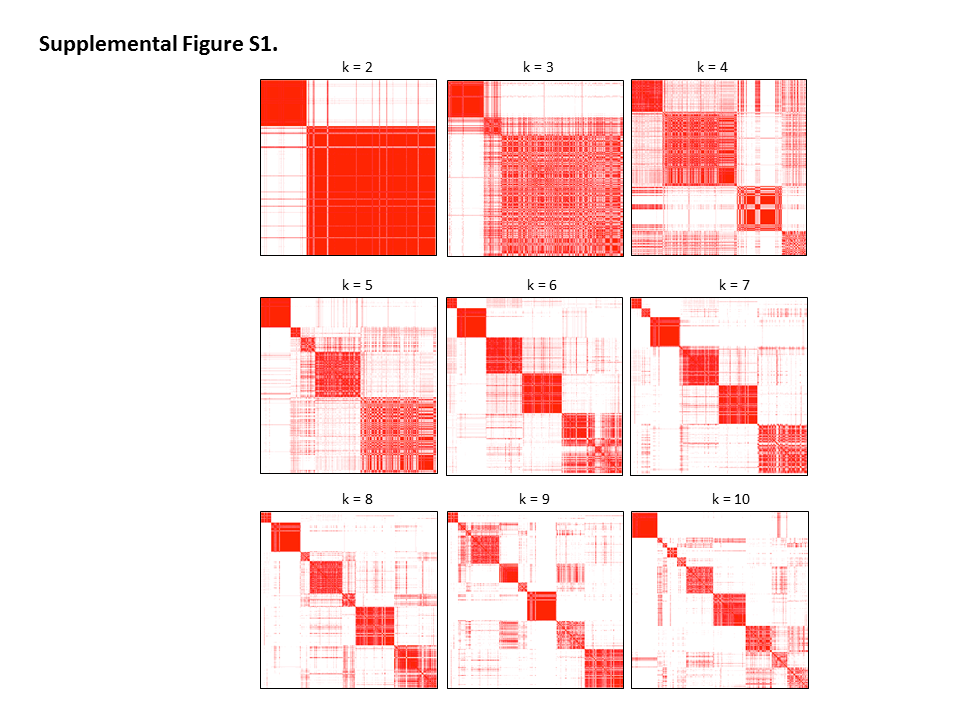

Supplement: Figure S1 — Consensus k-means heat maps for k = 2 to 10 generated with AKT pathway genes in the discovery dataset (GBM195). Red indicates total consensus (consensus index of 1) while white indicates no consensus (consensus index of 0). (TIF) [file pone.0100827.s001.tif]

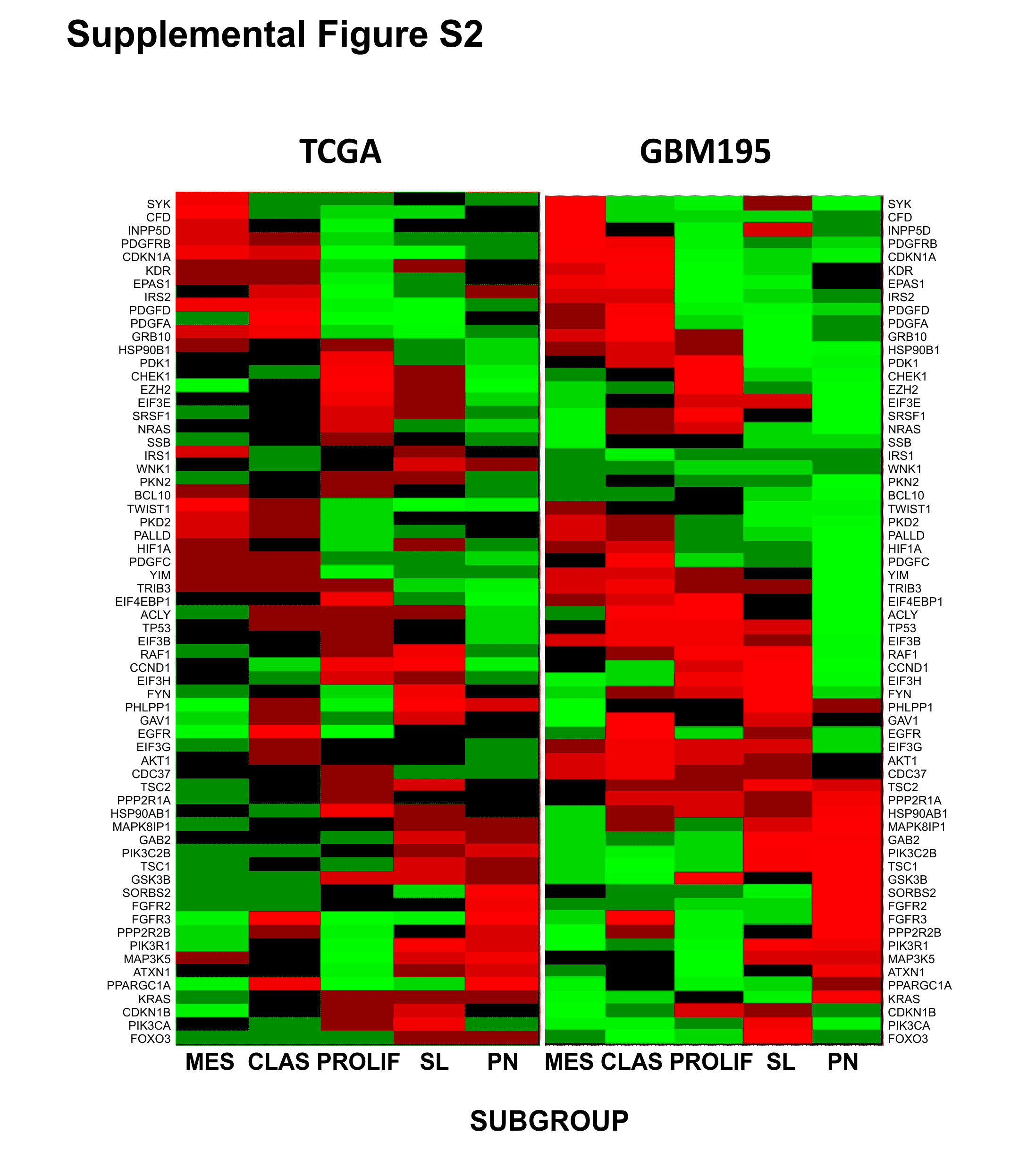

Supplement: Figure S2 — Average expression of AKT pathway genes in subgroups. Hierarchical clustering using AKT pathway genes was used to group GBM patients and genes in the discovery (GBM 195) dataset. Tumors in the validation dataset were grouped by AKT class keeping the same order of genes. The expression of AKT pathway genes in each class was averaged and is shown as a heatmap; red and green is high and low expression respectively. (TIF) [file pone.0100827.s002.tif]

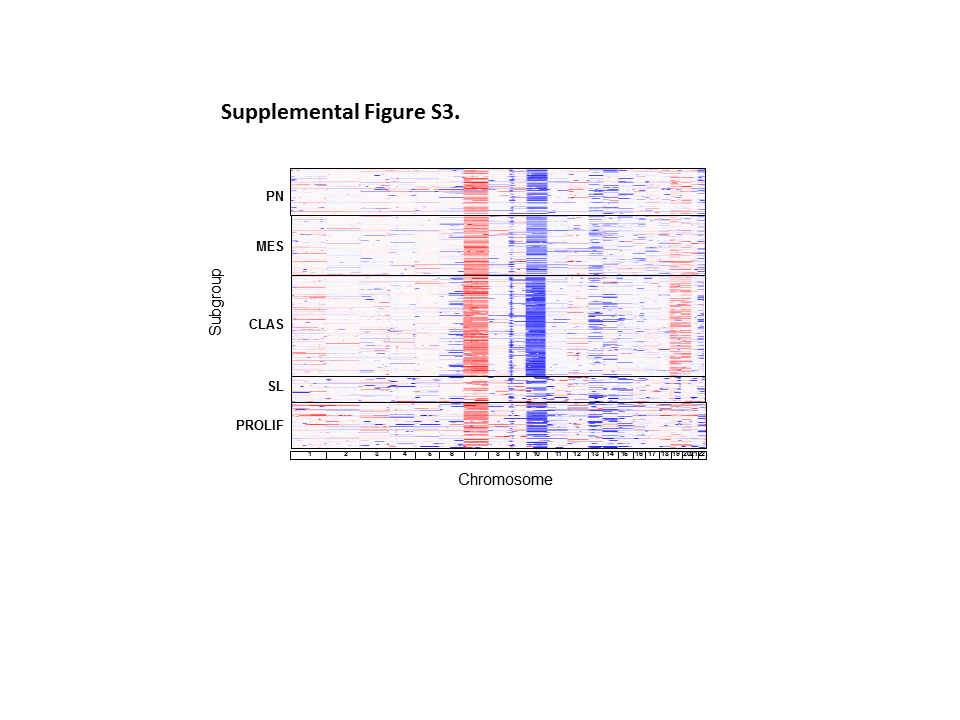

Supplement: Figure S3 — Log2 (tumor/normal) DNA copy number in subgroups. Amplifications (red) and deletions (blue) in subgroups (y axis) were determined by segmentation analysis of normalized signal intensities from TCGA SNP arrays using GISTIC and viewed with IGV by chromosomal location (x axis). (TIF) [file pone.0100827.s003.tif]
